# Identification of miRNA/FGFR2 Axis in Well-Differentiated Gastroenteropancreatic Neuroendocrine Tumors

**DOI:** 10.3390/ijms26157232

**Published:** 2025-07-26

**Authors:** Elisabetta Cavalcanti, Viviana Scalavino, Leonardo Vincenti, Emanuele Piccinno, Lucia De Marinis, Raffaele Armentano, Grazia Serino

**Affiliations:** 1 Department of Gastroenterology, National Institute of Gastroenterology IRCCS “S. de Bellis”, Research Hospital, Castellana Grotte, 70013 Bari, Italy; leonardo.vincenti@irccsdebellis.it; 2 Histopathology Unit, National Institute of Gastroenterology IRCCS “S. de Bellis”, Research Hospital, Via Turi 27, Castellana Grotte, 70013 Bari, Italy; lucia.demarinis@irccsdebellis.it (L.D.M.); raffaele.armentano@irccsdebellis.it (R.A.); 3 Laboratory of Molecular Medicine, National Institute of Gastroenterology IRCCS “S. de Bellis”, Via Turi 27, Castellana Grotte, 70013 Bari, Italy; viviana.scalavino@irccsdebellis.it (V.S.); emanuele.piccinno@irccsdebellis.it (E.P.)

**Keywords:** FGFR2, gastrointestinal neuroendocrine tumors, miRNAs

## Abstract

Gastroenteropancreatic neuroendocrine neoplasms (GEP-NENs) are rare tumors with different clinical and biological characteristics. Ki-67 staining and mitotic counts are the most commonly used prognostic markers, but these methods are time-consuming and lack reproducibility, highlighting the need for innovative approaches that improve histological evaluation and prognosis. In our previous study, we observed that the microRNA (miRNA) expression profile of GEP-NENs correlates with the three grades of GEP-NENs. This study aimed to characterize a group of miRNAs that discriminate well-differentiated GEP-NENs grading 1 (G1) and grading (G2). Fifty formalin-fixed and paraffin-embedded tissue specimens from well-differentiated GEP-NENs G1 and G2 tissues were used for this study. The expression levels of 21 miRNAs were examined using qRT-PCR, while FGFR2 and FGF1 protein expression were evaluated through immunohistochemistry (IHC). We identified four miRNAs (hsa-miR-133, hsa-miR-150-5p, hsa-miR-143-3p and hsa-miR-378a-3p) that are downregulated in G2 GEP-NENs compared to G1. Bioinformatic analysis revealed that these miRNAs play a key role in modulating the FGF/FGFR signaling pathway. Consistent with this observation, we found that fibroblast growth factor receptor 2 (FGFR2) expression is markedly higher in G2 NENs patients, whereas its expression remains low in G1 NENs. Our findings highlight the potential use of miRNAs to confirm the histological evaluation of GEP-NENs by employing them as biomarkers for improving histological evaluation and tumor classification.

## 1. Introduction

Gastroenteropancreatic neuroendocrine neoplasms (GEP-NENs) are a class of rare and heterogeneous tumors characterized by a broad spectrum of various clinical and biological characteristics [1]. In the last years, several studies have been conducted to gain a better understanding of NENs pathogenesis and refine grading systems and classification according to the site of origin, cell types, and pathological features [2]. Based on the World Health Organization (WHO) classification and the International Agency for Research on Cancer (IARC) classification, GEP-NENs are subdivided into well-differentiated tumors (NETs) and poorly-differentiated carcinomas (NECs) [3]. Moreover, GEP-NENs are classified by the grade of neuroendocrine differentiation and their proliferative index (mitotic count and/or Ki-67) from low-intermediate grade (G1–G2) to high grade (G3). According to this classification, G1 NETs are characterized by a mitotic count < 2/10 high power fields (HPFs) and/or a Ki-67 labeling index (LI) < 3%; G2 by a mitotic count 2–20/10 HPFs and/or a Ki-67 LI 3–20%; NECs are characterized by a mitotic count > 20/10 HPFs and/or a Ki-67 LI > 20% [3,4].

The absent or nonspecific symptoms implies that NENs are frequently diagnosed at an advanced, noncurative stage [5]. Currently, the survival rate for stage G1–G2 GEP-NENs is estimated to be 29% for the colon, 50% for the pancreas, and 69% for the small intestine [6]. Besides more accurate classification, there is a lack of specific markers for GEP-NEN diagnosis. To date, Chromogranin A (CgA), 5-Hydroxyindoleacetic Acid (5-HIAA), neuron specific enolase (NSE) synaptophysin (Syn), and cluster of differentiation 56 (CD56) (neural cell adhesion molecule) are widely used for diagnosis [7,8]. However, in GEP-NENs, these biomarkers lack sensitivity and specificity, resulting in a high-rate of false-positive [9].

Prognosis in NEN mainly depends on early diagnosis. In order to improve early diagnosis, intensive research activities have been established to identify novel and reliable biomarkers for clinical routine. In particular, the well-differentiated (WD) NENs (G1/G2) are much more common (70%) than the poorly differentiated (PD) NENs (G3) [10]. However, some WD cases exhibit a high level of proliferation whereas some poorly differentiated cases show a low mitotic activity [11]. In detail, well-differentiated neuroendocrine tumors (NEN) are characterized by slow tumor growth and the ability to secrete functionally active hormones. Moreover, by the time of diagnosis, more than 50% of patients have metastatic disease not amenable to curative surgery [12].

Over the years, considerable efforts have been made by researchers and clinicians to identify reliable biomarkers for the early detection of neuroendocrine tumors, with the goal of improving patient outcomes. However, the heterogeneity of these tumors has so far hindered the identification of a single analyte capable of encompassing the entire spectrum of neuroendocrine neoplasms.

Since their discovery, microRNAs (miRNAs) have been considered master modulators of gene expression, with unique miRNA signatures linked to various genetic, metabolic, infectious, and autoimmune diseases. miRNAs have been extensively studied in most common neoplasms, e.g., colorectal cancer, ovarian cancer, and lung cancer [13,14], but miRNAs in GEP-NENs are still poorly known. Recently, we identified the miRNAs expression profile of GEP-NENs that correlated with grading of GEP-NENs [15]. We defined the specific miRNAs signature for each GEP-NEN grade, identifying miRNAs that were commonly expressed in all GEP-NENs but at different levels. Among these, we studied the role of miR-96-5p, which showed higher expression levels from grade 1 to grade 3 and its correlation with FOXO1 expression.

In this study, we aimed to characterize a group of miRNAs that discriminate tumor stages of well-differentiated GEP-NENs G1 and G2. Starting from the published microarray analysis [15], we validated the expression of miRNAs differentially expressed in G1 and G2 GEP-NENs, correlating them with their gene targets.

## 2. Results

### 2.1. Clinicopathologic Features

Table 1 summarizes the main clinicopathologic features of the 50 patients enrolled in the study. The patients cohort included 23 females (46%) and 27 males (54%) with a median age of 63.5 years (range: 17–96). The most common primary site in our cohort of GEP-NEN patients was the small intestine (42%), followed by the stomach (34%), colon-rectum (14,%), pancreas (6%), appendix (2%) and gallbladder (2%). Of the 50 cases included, 25 were classified as grade 1 (50%) and 25 as grade 2 (50%), according to the 2010/2017 WHO classification [4]. Immune infiltration, particularly lymphocytic infiltration, was detected only in 7 (14%) of NEN G1/G2 tissue specimens. Then, curative treatments such as surgery or endoscopic resection were performed in 82% of patients (n = 41).

### 2.2. miRNA Expression Profile in Patients with G1 and G2 GEP-NENs

In our previous study, we defined the specific miRNA signature for each GEP-NEN grade, identifying miRNAs that are commonly expressed in all GEP-NEN but at different levels [15]. Here, starting from our microarray analysis [15], we analyzed the group of miRNAs that discriminate tumor stages GEP-NEN G1 and G2 (Table 2).

**Figure 1:**
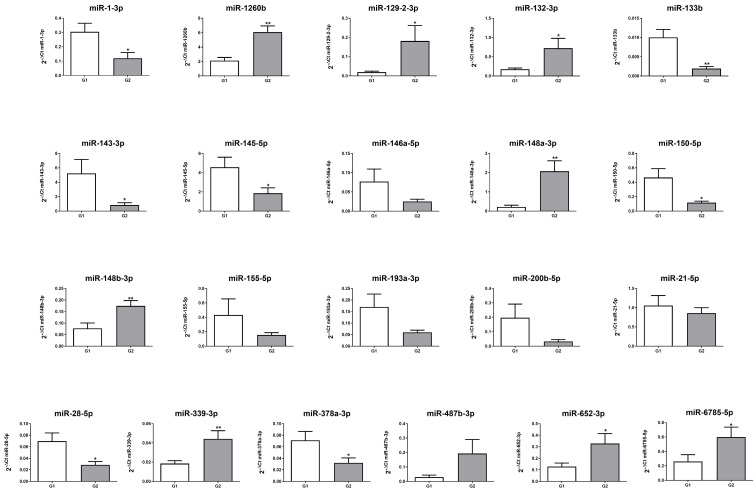
Differentially expressed miRNA in well-differentiated NENs comparing G1 (n = 10) and G2 (n = 10) groups, quantified using Real-Time PCR and expressed as 2^−ΔCt^. * *p* < 0.05 and ** *p* < 0.01.

In order to confirm the microarray results, we performed quantitative real-time PCR (qRT-PCR) for 21 miRNAs listed in Table 2 on miRNA samples isolated from an independent cohort of 20 GEP-NENs patients (10 G1 and 10 G2). The expression of 15 out of the 21 miRNAs was significantly modulated in G1 and G2 GEP-NENs patients (*p* < 0.05; Figure 1). Although a further 6 miRNAs confirmed the trend of the microarray, they did not achieve statistical significance (Figure 1).

### 2.3. In Silico Analysis of miRNA Targets

To investigate the molecular mechanisms underlying modulated miRNAs, we conducted bioinformatic analyses to predict their target genes. As evidenced by bioinformatic analyses, deregulated miRNAs were found to be involved in many characteristic pathways of cancer. In particular, the modulated miRNAs are involved in key pathways of cancer onset such as ECM-receptor interaction, TGF-beta signaling, MAPK signaling, Pathways in cancer, Proteoglycans in cancer (Table S1). Interestingly, some of these downregulated miRNAs (hsa-miR-133b, hsa-miR-150-5p, hsa-miR143-3p, hsa-miR-378a-3p) modulated key components of the FGF/FGFR signaling pathway such as FGFR2, FGF1 and FGF2.

### 2.4. FGFR2 and FGF1 Protein Expression in GEP-NENs

**Figure 2:**
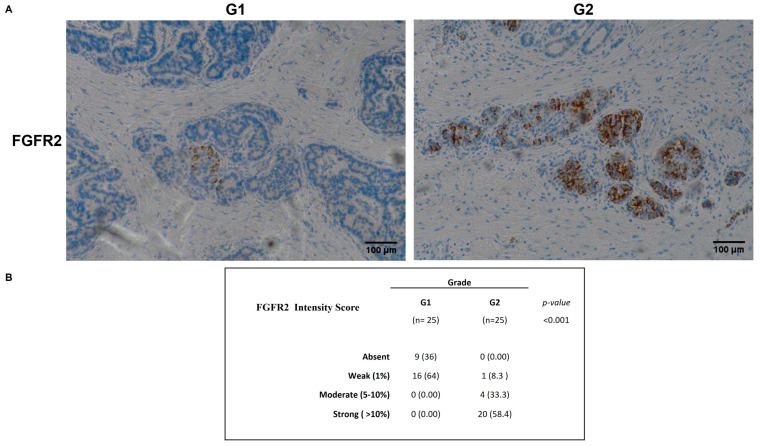
FGFR2 staining intensity on neoplastic cells differed between G1 and G2 tumor grades. (**A**) Weak FGFR2 staining (1+) in a G1 patient and strong FGFR2 staining (3+) in a G2 patient. (**B**) FGRF2 intensity score in G1 and G2 GEP-NENs patients.

To evaluate the biological significance of miRNA associated with G1/G2 NENs, we analyzed the FGFR2 and FGF1 tissue expression in 50 NENs GEP-NENs tissue by IHC. FGF1 positive staining was primarily observed as cytoplasmic or nuclear immunoreactivity, whereas FGFR2 positive staining was mainly detected in the stromal compartment. No significant correlation was found between the expression levels of FGFR2 and FGF1 and patients gender, age, tumor size, or lymph node metastasis. Notably, we highlighted a significant correlation between protein expression of FGFR2 and grading of GEP-NENs (*p* = 0.001). In particular, FGFR2 staining intensity on neoplastic cells differed between G1 and G2 tumor grades (Figure 2A). Based on FGFR2 signal intensity, we developed a scoring system ranging from 0 to 3+ (Figure 2B), indicating absent to strong staining. This score differed between G1 and G2 tumors. Specifically, among G1 patients, none showed strong FGFR2 staining (3+; 0%), 16 cases (64%) showed weak staining (1+), and 9 cases (36%) showed no detectable staining (0+).

**Figure 3:**
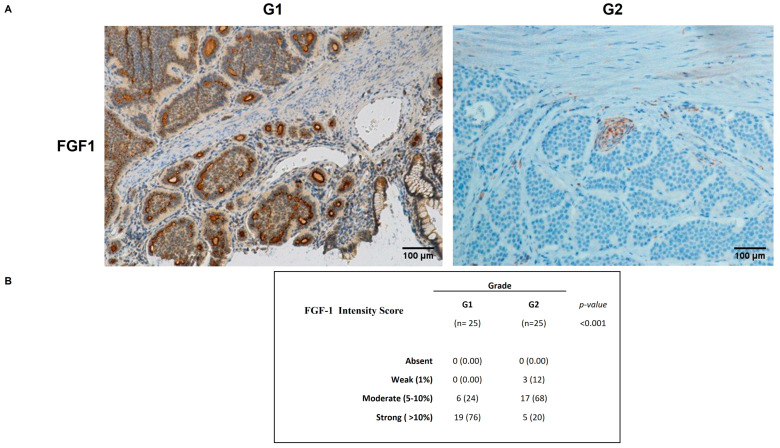
Tissue-specific expression patterns of FGF1. (**A**) FGF1 tissue expression was significantly stronger in G1 cases. Moderate positivity FGF1 staining was found in G2 cases. (**B**) FGF1 intensity score in G1 and G2 GEP-NENs patients.

FGF1 tissue expression was significantly stronger in G1 than in G2 (Figure 3A). Importantly, 76% of G1 cases showed a strong positivity and cytoplasmic staining for FGF1 (3+). Inversely, 68% of G2 cases showed a moderate positivity staining for FGF1 (2+) and only 20% cases showed strong FGF1 staining (3+), suggesting it has a peculiar functional role (Figure 3B).

## 3. Discussion

GEP-NENs are a group of tumors with similar phenotypes but different in origin, morphology, molecular profile, type- and site-specific prognosis, aggressiveness, and response to treatment. Despite the new classification criteria defined by the WHO (2010/2017 WHO), the clinical behavior of NEN is highly unpredictable so that the low-grade tumor cases are often unexpectedly diagnosed at an advanced disease stage [16,17]. At present, it is not yet possible to define which biological, pathological or clinical characteristics can reliably characterize the subset of patients with ‘low risk’ within the G1–G2 grading. In this era of personalized medicine, biomarkers predictive of therapy response are central to treatment decision making. Therefore, the hallmarks of cancers including differentiation, anaplasia, proliferative capacity and metastatic tendencies, angiogenesis and immunosuppression are being paid increasing attention by researchers [18].

Epigenetic alterations—such as DNA methylation, histone modifications and miRNAs—hold significant promise for improving cancer diagnostics and guiding precision medicine [19,20], and can enhance diagnostic accuracy and subclassification of tumors [21]. Moreover, the identification of epigenetically dysregulated pathways opens avenues for targeted therapies that specifically interfere with these reversible modifications, offering new strategies for the treatment of resistant or aggressive cancers [22]. Differential methylations of specific gene promoters were associated with GEP-NENs [23]. Mutations in chromatin regulators such as MEN1, ATRX, and DAXX are commonly observed in NENs and contribute to epigenomic instability [24]. Many studies have focused on the potential of miRNAs as molecular biomarkers able to improve tumor grade stratification, grading and tissue discrimination in GEP-NENs [15,25,26,27]. The miRNAs expression patterns exhibit distinct correlations with tumor differentiation, proliferation rates, and metastasis. In fact, in our previous work, we demonstrated that the miRNA expression profile of GEP-NENs was correlated with grading of GEP-NENs. Moreover, we studied the role of miR-96-5p that showed raised expression levels from grade 1 to grade 3 and its correlation with FOXO1 expression [15].

In this study, we have identified a group of miRNAs that distinguish well-differentiated G1 and G2 GEP-NENs. Moreover, through bioinformatic analysis, we demonstrated that these deregulated miRNAs played a crucial role in the regulation of many pathways associated with cancer progression. Interestingly, some miRNAs found to be downregulated in well-differentiated G2 GEP-NENs (hsa-miR-133, hsa-miR-150-5p, hsa-miR143-3p, hsa-miR-378a-3p) regulated key components of the FGF/FGFR signaling pathway, such as FGFR2 and FGF1. Their downregulation can affect critical processes including angiogenesis, wound healing, and cancer progression.

NENs are characterized by exceptionally high vascularization, reflecting the structure of normal endocrine glands [28]. Well-differentiated NENs exhibit extensive neovascularization despite low endothelial proliferation, whereas poorly differentiated carcinomas display reduced microvascular density [29,30]. Unlike in most carcinomas, high vessel density in NENs is linked to a better prognosis, longer survival and increased VEGF expression—a phenomenon known as the “neuroendocrine paradox” [29,31]. As tumor aggressiveness increases, vascularization typically decreases, contrasting with the vessel-rich architecture of normal tissue. Key angiogenic pathways such as VEGF, FGF (fibroblast growth factor), and PDGF (platelet-derived growth factor) are involved in NEN development and hold clinical relevance [32,33]. Our previous research showed that PDGFRα expression is linked to the morphology and vascular features of low-grade NENs, correlating with lower microvessel density and reduced tumor growth. This suggests that PDGFRα may play a central role in epithelial–stromal interactions and influence both tumor and stromal cell behavior through paracrine and possibly autocrine mechanisms [34].

The FGF/FGFR system also regulates key cellular processes, such as proliferation, differentiation, cell migration [35], and its dysregulation has been linked to cancer development and inducing resistance to chemotherapy, including NENs [36,37]. The FGF/FGFR axis is a critical driver of VEGF-independent revascularization in pNETs and can therefore mediate evasive resistance to antiangiogenic therapy [38]. Several studies underline its function in the modulation of tumor fibrosis, proliferation, angiogenesis and drug resistance, through a dynamic cross talk between NEN cells, fibroblasts, endothelial cells and inflammatory cells [39]. Fibroblast growth factor has a key role in maintaining tumor angiogenesis and the inhibition of the FGF/FGFR axis suppresses neoangiogenesis and tumor growth [40]. Fibroblast growth factor-1 (FGF1) and fibroblast growth factor-2 (FGF2) are expressed in approximately 40% and 100% of GEP-NENs, respectively, while fibroblast growth factor receptor (FGFR) 1–4 are expressed by 68–88% of these malignancies [41,42]. La Rosa et al. identified cytoplasmic immunoreactivity for FGF1 in 26 (43%) of 60 GEP-NENs [42]. They also noted that FGFR expression is rare in normal gut endocrine cells, suggesting that the FGF/FGFR system does not need autocrine function to regulate endocrine cell activity in normal mucosa.

In this study, we demonstrated that FGFR2 is highly expressed in G2 NENs, while it is weakly expressed in G1 NENs. G2 NENs establish a pro-angiogenic microenvironment via molecular signals and cellular adaptations that are comparatively less pronounced in G1 NENs. G2- NENs, being more aggressive and faster-growing than G1 tumors, must therefore develop angiogenic capabilities to avoid necrosis and continue growing. FGFR2 immunoreactivity was related to the Ki-67 proliferative index and malignancy of the endocrine tumors investigated. The findings indicate that FGFR2 was involved in tumor development and progression.

The FGF system also appears to be also involved in the mechanism of gastrointestinal NEN fibrosis. Our results demonstrated a correlation between FGF1 and the amount of fibrous stroma in G1 NENs. Furthermore, FGF1 was significantly more highly expressed in G1 NENs, while it was moderately expressed in G2 NENs.

The positive diffuse stromal immunostaining for FGF1 suggested a possible functional role for FGF1 secreted by tumor cells in the proliferation of stromal fibroblasts or other mesenchymal cell types. Probably FGF1 is highly expressed in G1 NENs because it is associated with differentiation and maintenance functions, an assumption which is consistent with the more benign and regulated nature of these tumors. In contrast, G2 NENs tend to express pro-angiogenic and pro-proliferative factors such as FGFR2. Moreover, G1 and G2 NENs activate different molecular pathways. It is possible that G1 NENs rely more on FGF1 to trigger signaling pathways involved in differentiation or endocrine function, while G2 NENs preferentially utilize ligands like FGF2 or activate FGFR2, which are linked to cell proliferation and angiogenesis. Future studies are needed to evaluate whether the co-expression of FGF1 and FGFR2 may characterize a specific tumor subtype, such as FGFR2-amplified gastric tumors or aggressive neuroendocrine tumors.

## 4. Materials and Methods

### 4.1. Patients Characteristics and Pathological Assessment

Fifty formalin-fixed and paraffin-embedded tissue specimens of NENs collected from January 2020 to January 2023 at the IRCCS “Saverio De Bellis” of Castellana Grotte (Ba, Italy) were included in this study after approval by the local Ethical Committee. Only adult patients (aged 18 years or older) were considered eligible for inclusion. Additional inclusion criteria were the availability of sufficient tumor tissue, formalin-fixed and paraffin-embedded (FFPE), suitable for molecular analyses, including RNA extraction. Exclusion criteria comprised patients diagnosed with poorly differentiated neuroendocrine carcinomas or mixed neuroendocrine–non-neuroendocrine neoplasms, those with inadequate tissue samples in terms of quality or quantity, as well as individuals affected by other concurrent malignancies, except for non-melanoma skin cancers or in situ cervical cancer. The following clinicopathological characteristics were collected for all patients: age, gender, primary site, tumor grade and metastasis (Table 1).

Histology was assessed in all tumors by two pathologists who reviewed FFPE tissue sections stained with hematoxylin and eosin (H&E), and a representative paraffin block from each specimen was chosen for IHC analysis. On H&E and PAS mucin-stained sections, the cytological characteristics of cells, the presence of ulcerations, perineural infiltration, vascular permeation necrosis and lymph node metastasis were evaluated. All the cases were reviewed to confirm the diagnoses according to the WHO 2010 criteria, and for pNETs, according to the last WHO 2017 classification [4].

### 4.2. miRNAs Isolation

Total RNA, including small RNA fraction, was isolated from FFPE sections, at 5 µm-thickness using the miRNeasy FFPE kit (Qiagen, Hilden, Germany), according to the manufacturer’s protocol including the treatment of sections with Deparaffinization Solution (Qiagen, Hilden, Germany). Total RNA was then eluted in ribonuclease-free water. The RNA concentration was determined with NanoDrop ND-2000 Spectrophotometer (Thermo Fisher Scientific, Waltham, MA, USA).

### 4.3. Quantitative Real-Time PCR

Total RNA, including small RNA, was reverse transcribed with the TaqMan Advanced miRNA cDNA Synthesis Kit (Thermo Fisher Scientific, Waltham, MA, USA) using the manufacturer’s protocol. Real-time RT-PCR for the quantification of 21 miRNAs (hsa-miR-1-3p, hsa-miR-1260b, hsa-miR-129-2-3p, hsa-miR-132-3p, hsa-miR-133b, hsa-miR-143-3p, hsa-miR-145-5p, hsa-miR-146a-5p, hsa-miR-148a-3p, hsa-miR-148b-3p, hsa-miR-150-5p, hsa-miR-155-5p, hsa-miR-193a-3p, hsa-miR-200b-5p, hsa-miR-21-5p, hsa-miR-28-5p, hsa-miR-339-3p, hsa-miR-378a-3p, hsa-miR-487b-3p, hsa-miR-652-3p, hsa-miR-6785-5p) was performed with TaqMan Advanced miRNA assays and TaqMan Fast Advanced Master mix (Thermo Fisher Scientific, Waltham, MA, USA). miRNAs expression data were normalized on the geometric mean of miR-26a-5p and hsa-miR-320a. Real-time PCR amplification reactions were performed in 20 µL of final volume on a CFX96 System (Biorad Laboratories, Hercules, CA, USA) using the RT-PCR protocol below: 50 °C for 2 min, 95 °C for 20 s, followed by 40 cycles of 95 °C for 3 s and 60 °C for 30 s.

### 4.4. IHC and IHC Evaluation

IHC analysis for FGFR2 and FGF1 was performed on the FFPE of 50 patients with GEP-NENs. Tumor sections of 4 µm were freshly cut and dried at 60 °C for 30 min. IHC analysis was carried out in sections after deparaffinization for 30 min and then rehydration in grades of alcohol. Antigen retrieval was performed at 90 °C for 20 min with Tris-borate-EDTA Buffer. To assess the FGFR2 and FGF1 staining employed for the present study, antibodies (FGF1, clone PAS 79249, Invitrogen, Carlsbad, CA, USA at 1:200 dilution; FGFR2 clone AMab235277, Abcam, Cambridge, UK at 1:100 dilution) were evaluated on the NENs, using an automated autostainer (cat. K5007, Dako, Glostrup, Denmark). The Real Envision DAB Substrate Kit (DAKO) was used according to the manufacturer’s instructions. FGFR2 and FGF1 expression were scored for all staining patterns, according to both the staining intensity and the percentage of positively stained cells, by two independent, blinded pathologists. The proportion of FGFR2 and FGF1 positive cells were estimated as the percentage of total tumor cells; tumor cells typically showed cytoplasmatic staining with a variable nuclear staining component. FGFR2 and FGF1 expression levels were scored as 0: (no staining) negative; 1: weak expression, but weaker than the positive control, staining in <5% of tumor cells; 2: moderate expression in 5–10% of tumor cells; and 3: strong more than positive control staining in >10% of the tumor cells. For the data assessment, our cases were considered positive for FGFR2 and FGF1 expression only if they had scores of 2+ or 3+.

### 4.5. Statistical and Bioinformatical Analyses

miRNA targets were predicted using the databases miRBase 21.1 (https://www.mirbase.org/ (accessed on 12 March 2024)) [43], TargetScan 7.1 (http://www.targetscan.org/vert_71/ (accessed on 12 March 2024)) [44], miRWalk 2.0 (http://zmf.umm.uni-heidelberg.de/apps/zmf/mirwalk2/ (accessed on 12 March 2024)) [45], and TarBase v.8 (http://carolina.imis.athena-innovation.gr/diana_tools/web/index.php?r=tarbasev8/index (accessed on 13 March 2024)) [46]. Potential targets were identified based on overlapping results from the five algorithms and selecting targets genes predicted by at least two of the algorithms. To assess biologic relationships among genes controlled by deregulated miRNAs, we used miRSystem ver. 20160513 (https://mirsystem.cgm.ntu.edu.tw/ (accessed on 21 March 2024)) [47] and miRWalk (http://mirwalk.umm.uni-heidelberg.de/ (accessed on 21 March 2024)) [45] software.

Statistical analysis was performed using GraphPad Prism statistical software 10.0.2. Statistical differences between different conditions were assessed with two-tailed Student’s *t* test. All values are expressed as the mean ± SEM. Results were considered statistically significant at *p* < 0.05.

## 5. Conclusions

In conclusion, this study examined the miRNAs expression profile that discriminates tumor stages of well-differentiated GEP-NENs G1 and G2. We identified four downregulated miRNAs (hsa-miR-133, hsa-miR-150-5p, hsa-miR143-3p, hsa-miR-378a-3p) in G2 GEP-NENs compared to G1 that play a crucial role in regulating the FGF/FGFR signaling pathway. In alignment with this finding, we observed a differential expression of FGFR2 protein between GEP-NENs of the two different histological grades. Specifically, well-differentiated G1 tumors exhibited low or absent FGFR2 expression, in line with their lower proliferative activity and more indolent clinical behavior. In contrast, G2 tumors showed a higher level of FGFR2 expression, which may reflect increased activation of signaling pathways involved in tumor proliferation and progression. This difference has important clinical implications. The increased FGFR2 expression observed in G2 tumors suggests a potential role for FGFR2 as both a prognostic biomarker associated with a more aggressive disease course and as a therapeutic target. Indeed, FGFR inhibitors currently under investigation in other tumor types may offer a promising treatment strategy for GEP-NENs, particularly for G2 tumors with high FGFR2 expression. Although the direct expression of FGF1 and FGFR2 in NENs is not widely documented, the FGF/FGFR system could play a significant role in the biology of these tumors and may offer new targeted therapeutic approaches. In addition, the expression patterns of the identified miRNAs, in combination with FGFR pathway biomarkers offer valuable insights for refining tumor classification and driving therapeutic decisions, highlighting their importance in personalized treatment strategies for GEP-NEN patients. The slight limitations of this study must be noted. Due to the small sample size of patients, other studies are needed to confirm the identified biomarkers in a larger multicenter prospective cohort of GEP-NEN patients. Furthermore, since the data were derived from tissue samples, future studies should explore whether these biomarkers can be identified in blood samples to facilitate a non-invasive classification of GEP-NEN.

## Figures and Tables

**Table 1:** Clinicopathologic features of 50 patients with GEP -NEN.

	Tot	%
**Gender**		
Male	27	54
Women	23	46
**Age, years**		
Median	63.5	
**Tumor site**		
Stomach	17	34
Small Intestinal	21	42
Colon-rectum	7	14
Appendix vermiformis	1	2
Pancreas	3	6
Gallbladder	1	2
**Grade WHO classification**		
G1	25	50
G2	25	50
**Angioinvasion**		
Absent	25	83
Present	5	17
**Lymphocytic infiltration**		
Absent	43	86
Present	7	14
**Perineural infiltration**	Yes	40
**Vascular permeation**	Yes	13
**Necrosis**	Yes	11

**Table 2:** List of miRNAs differentially expressed between G1 and G2 GEP-NENs.

ID miRNA	Expression G2 vs. G1
hsa-miR-1-3p	down
hsa-miR-1260b	up
hsa-miR-129-2-3p	up
hsa-miR-132-3p	up
hsa-miR-133b	down
hsa-miR-143-3p	down
hsa-miR-145-5p	down
hsa-miR-146a-5p	down
hsa-miR-148a-3p	up
hsa-miR-148b-3p	up
hsa-miR-150-5p	down
hsa-miR-155-5p	down
hsa-miR-193a-3p	down
hsa-miR-200b-5p	down
hsa-miR-21-5p	down
hsa-miR-28-5p	down
hsa-miR-339-3p	up
hsa-miR-378a-3p	down
hsa-miR-487b-3p	up
hsa-miR-652-3p	up
hsa-miR-6785-5p	up

## Data Availability

Data are within the article and Supplementary Materials.

## Supplementary Materials

The following supporting information can be downloaded at: https://www.mdpi.com/article/10.3390/ijms26157232/s1

## Abbreviations

NENs	Neuroendocrine neoplasms
GEP-NENs	Gastroenteropancreatic neuroendocrine neoplasms
WHO	World Health Organization
IARC	International Agency for Research on Cancer
Ki-67	Antigen Kiel 67
HPFs	high power fields
LI	labeling index
CgA	Chromogranin A
5-HIAA	5-Hydroxyindoleacetic Acid
NSE	neuron specific enolase
Syn	synaptophysin
CD56	cluster of differentiation 56
miRNA	microRNA
qRT-PCR	quantitative real-time PCR
ECM	extracellular cell matrix
TGF	Transforming Growth Factor
MAPK	mitogen-activated protein kinase
FGFR2	fibroblast growth factor receptor 2
FGF1	fibroblast growth factor 1
IHC	immunohistochemistry
VEGF	Vascular endothelial growth factor
H&E	hematoxylin and eosin
PAS	Periodic acid–Schiff
DAB	3,3′-Diaminobenzidina
